# GPRC5A Is a Negative Regulator of the Pro-Survival PI3K/Akt Signaling Pathway in Triple-Negative Breast Cancer

**DOI:** 10.3389/fonc.2020.624493

**Published:** 2021-02-16

**Authors:** Lu Yang, Shaorong Zhao, Tong Zhu, Jin Zhang

**Affiliations:** ^1^The Third Department of Breast Cancer, Tianjin Medical University Cancer Institute and Hospital, National Clinical Research Center for Cancer, Tianjin, China; ^2^Key Laboratory of Cancer Prevention and Therapy, Tianjin Medical University Cancer Institute and Hospital, Tianjin, China; ^3^Tianjin’s Clinical Research Center for Cancer, Tianjin Medical University Cancer Institute and Hospital, Tianjin, China; ^4^Key Laboratory of Breast Cancer Prevention and Therapy, Tianjin Medical University, Ministry of Education, Tianjin, China

**Keywords:** G-protein-coupled receptor family C, member 5, group A (Gprc5a), cell apoptosis, PI3K/Akt, breast cancer, triple-negative breast cancer (TNBC)

## Abstract

Breast cancer is one of the most common types of malignancy worldwide; however, its underlying mechanisms remain unclear. In the present study, we investigated the roles of G-protein-coupled receptor family C, member 5, group A (GPRC5A) in cell apoptosis in triple-negative breast cancer (TNBC). The expression of GPRC5A in breast cancer cell lines was detected by real time PCR and western blot. And the results suggested that GPRC5A was downregulated in breast cancer cell lines compared to normal breast epithelial cell lines. Additionally, the expression of GPRC5A in TCGA database was analyzed *in silico*. GPRC5A exhibited the lowest expression levels in TNBC compared to ER^+^ and HER2^+^ breast cancer. Overexpression of GPRC5A in MDA-MB-231 and MDA-MB-468 cells promoted apoptosis, whereas depletion of GPRC5A in T47D and MCF7 cells inhibited cell apoptosis *via* the intrinsic apoptotic pathway. We performed RNA-sequencing in GPRC5A overexpressed MDA-MB-231 and the control cells. The results facilitated the identification of a number of signaling pathways involved in this process, and the PI3K/Akt signaling pathway was found to be one the most important. A specific activator of the PI3K/Akt signaling pathway inhibited apoptosis of breast cancer cells, whereas cotreatment of this activator with a GPRC5A-expressing plasmid reduced this effect. Similarly, a specific inhibitor of the PI3K/Akt signaling pathway increased cell apoptosis by activating caspase-3 and caspase-9, whereas co-incubation of the inhibitor with a short hairpin RNA targeting GPRC5A significantly reduced the cell apoptotic rate. Additionally, the overexpression of GPRC5A suppressed tumor growth by inducing cell apoptosis *in vivo*. Taken together, the present study identified GPRC5A as a protective factor against the progression of human triple-negative breast cancer by increasing cell apoptosis *via* the regulation of the PI3K/Akt signaling pathway.

## Introduction

Breast cancer is one of the most common diseases among females, with ~200,000 new cases diagnosed every year worldwide, and is a major cause of mortality in females despite age or ethnicity. Triple-negative breast cancer (TNBC) is the most challenging subtype of breast cancer to treat. TNBC refers to breast cancer where the genes encoding estrogen receptor (ER), progesterone receptor and human epidermal growth factor receptor 2 are not upregulated (1). TNBC is poorly differentiated and can migrate and proliferate in distant locations faster compared with other types of breast cancer, which leads to a worse prognosis and a short 5-year survival rate (2, 3). The lack of targetable receptors in TNBC makes targeted therapy an important topic of research and a number of studies are trying to identify novel markers for the treatment and diagnosis of breast cancer.

G-protein-coupled receptor family C, member 5, group A (GPRC5A), also termed retinoic acid-inducible 3, belongs to the largest protein superfamily containing >700 genes within the human genome (4, 5). GPRC5A was first identified in 1998 in numerous types of human cancer, including colon cancer (6), colorectal cancer (7), and pancreatic cancer (8). A high prevalence of GPRC5A germline mutations have been identified in BRCA1-mutant breast cancer (9). Furthermore, GPRC5A has been demonstrated to be a potential therapeutic target (10), and can inhibit epidermal growth factor receptor and its downstream pathway (11). However, the detailed mechanisms underlying the regulatory roles of GPRC5A remain unclear.

Cell apoptosis is a form of programmed cell death that occurs in multicellular organisms (12) and serves significant roles in various types of cancer (13). Our previous researches implicate GPRC5A as a tumor suppressor in breast cancer cells, and GPRC5A exerts its tumor-suppressive function by inhibiting EGFR related cell proliferation. Meanwhile, we found GPRC5A as a tumor suppressor has effect on breast cancer cell apoptosis (11). Thus, the present study aimed to examine whether the role of GPRC5A is associated with cell apoptosis. Cultured breast cancer cells were used in the present study to detect the expression of GPRC5A. RNA-sequencing (RNA-seq) analysis was performed to investigate the detailed mechanism underlying the protective role of GPRC5A. A specific activator and an inhibitor of the PI3K/Akt signaling pathway were also used in the presence or absence of a GPRC5A expressing plasmid or short hairpin RNA (shRNA) targeting GPRC5A (shGPRC5A). The present results suggested a protective role of GPRC5A in human breast cancer and investigated its underlying mechanism, facilitating the development of novel clinical treatments for patients with TNBC.

## Materials and Methods

Cell Culture and Transfection. The human breast cancer cell lines T47D, MDA-MB-231, MDA-MB-468, MCF7, and SK-BR-3 were purchased from the Cell Bank of Shanghai Biological Institute, Chinese Academy of Science. The breast epithelial cell line MCF10A was obtained from the American Type Culture Collection and was used as control cell line to assess the expression level of GPRC5A. All cell lines were cultured in DMEM (Gibco; Thermo Fisher Scientific, Inc.) supplemented with 10% FBS (Gibco; Thermo Fisher Scientific, Inc.) and maintained at 37˚C with 5% CO_2_.

Specific shRNAs against GPRC5A were designed and synthesized by Shanghai GenePharm Co., Ltd. and dissolved in 20 µM. The GPRC5A-expressing plasmid was cloned by PCR with *Xho*I and *Hind*III restriction enzymes into pcDNA3.1 and validated by DNA sequencing. Cell transfection was performed with Lipofectamine^®^ 3000 (Invitrogen; Thermo Fisher Scientific, Inc.), according to the manufacturer’s protocol. Cells transfected with empty vector or a non-targeting shRNA were used as controls.

SF1670, a specific PTEN inhibitor, and deguelin were used as the activator and inhibitor of the PI3K/Akt signaling pathway, respectively, and both were obtained from Selleck Chemicals and used at a final concentration of 10 µM.

Reverse Transcription-Quantitative PCR. Total RNA from cultured breast cancer cells was extracted using TRIzol^®^ reagent (Invitrogen; Thermo Fisher Scientific, Inc.) and quantified with Nanodrop 2000 by measuring the absorbance at 260 and 280 nm. A total of 1 µg RNA was reverse transcribed into cDNA by Prime Script Master mix (Takara Biotechnology Co., Ltd.) according to the manufacturer’s protocol. qPCR was performed with SYBR Premix EX Taq TM II (Takara Biotechnology Co., Ltd.) on an ABI 7900 qPCR detection system (Thermo Fisher Scientific, Inc.) with the following conditions: Initial denaturation at 95˚C for 5 min, followed by 45 cycles of 10 s at 95˚C (denaturation), 10 s at 60˚C (primer annealing), and 10 s at 72˚C (elongation), and a final extension step for 10 min at 72˚C. GAPDH was used as the internal reference. Relative mRNA expression levels were calculated using the 2^-ΔΔCq^ method. The following primer sequences were used for the qPCR analysis: GPRC5A forward, 5’-ATGGCTACAACAGTCCCTGAT-3’ and reverse, 5’-CCACCGTTTCTAGGACGATGC-3’; PI3K forward, 5’-GTCCTATTGTCGTGCATGTGG-3’ and reverse, 5’-TGGGTTCTCCCAATTCAACC-3’; Akt forward, 5’-TTCTATGGCGCTGAGATTGTGT-3’ and reverse, 5’-GCCGTAGTCATTGTCCTCCAG-3’; mTOR forward, 5’-ATGCTTGGAACCGGACCTG-3’ and reverse, 5’-TCTTGACTCATCTCTCGGAGTT-3’; and GAPDH forward, 5’-GTGGACATCCGCAAAGAC-3’ and reverse, 5’-AAAGGGTGTAACGCAACTA-3’.

Vector Construction, Virus Production, and the Construction of Stable Cell Lines. The GPRC5A plasmid was purchased in HANHENG Co. (China). Then GPRC5A were amplified by reverse transcription PCR and then inserted into pLV-cDNA-MCS-bsd cloning vector (Biosettia). Lentivirus stocks were produced by transfecting a 4-plasmid system from 293T in accordance with the manufacturer’s instruction (Biosettia). The lentivirus stocks were added to MDA-MB-231 cell line for 16h. After 48h, 10 μg/ml blasticidin S was added for 3 days to obtained MDA-MB-231-GPRC5A cell lines.

Cell Apoptosis Assays. The annexin V/propidium iodide (PI) assay was performed according to the manufacturer’s protocol (Invitrogen; Thermo Fisher Scientific, Inc.). Briefly, MDA-MB-231 and MDA-MB-468 cells were transfected with GPRC5A-expressing plasmid and treated with or without SF1670. In addition, T47D and MCF7 cells were transfected with shGPRC5A in the presence or absence of deguelin. Subsequently, cells were washed twice with pre-cold PBS, trypsinized and re-suspended in 100 μl binding buffer with 2.5 μl FITC-conjugated annexin-V and 1 μl PI (100 μg/ml). Cells were then incubated at room temperature for 15 min in the dark. A total of ~10,000 cells were collected and analyzed with a flow cytometer.

Relative Caspase Activities. The relative activities of caspase-3, caspase-8, and caspase-9 were determined using caspase activity kits (cat. nos. C1115, C1151, and C1157, respectively; Beyotime Institute of Biotechnology), according to the manufacturer’s protocols. Briefly, MDA-MB-231 and MDA-MB-468 cells were transfected with GPRC5A expressing plasmid with or without SF1670, and T47D and MCF7 cells were treated with shGPRC5A in the presence or absence of deguelin. Subsequently, cell lysates were collected by low speed centrifugation at 1,000 x g for 5 min at 4°C. An equal amount of protein (10 µl) was added into 96-well plates in triplicate and mixed with 80 μl reaction buffer supplemented with caspase substrates (2 mM). Following incubation at 37°C for 4 h, caspase activities were determined at an absorbance of 450 nm.

Western Blot Assays. Cells were transfected with shGPRC5A or GPRC5A-expressing plasmid for 48 h and then lysed with RIPA lysis buffer (Beyotime Institute of Biotechnology) and fresh protease inhibitor cocktail (Thermo Fisher Scientific, Inc.). The protein concentration was assessed using a bicinchoninic assay kit (Thermo Fisher Scientific, Inc.), according to the manufacturer’s protocol. A total of 40 µg protein extract was loaded into each well of a 12% SDS-PAGE gel and transferred to a nitrocellulose membrane (EMD Millipore). Following blocking with 5% milk, the membranes were incubated with primary antibodies against GPRC5A (cat. no. CSB-PA818781DSR1HU; Cusabio Biotech Co., Ltd.), caspase-3 (cat. no. ab13585; Abcam), caspase-9 (cat. no. ab2324; Abcam), cytochrome C (cat. no. ab13575; Abcam) and GAPDH (cat. no. sc-32233; Santa Cruz Biotechnology, Inc.) overnight. The secondary antibodies were purchased from Santa Cruz Biotechnology, Inc. All antibodies were diluted at a ratio of 1:1,000. Subsequently, proteins were detected using an enhanced chemiluminescence kit (EMD Millipore). The immunoreactive bands were quantified by densitometry with ImageJ software (National Institutes of Health).

RNA-Seq Analysis. MDA-MB-231 cells were transfected with or without GPRC5A expressing plasmid for 48 h and total RNA was then extracted for RNA-seq in triplicate. RNA-seq was performed and analyzed by AnNuoNeng Co. Different signaling pathways were investigated and the significant differentially expressed genes (P*<*0.05) were classified into corresponding signaling pathways.

Tumor Xenograft. The female NOD/SCID mice at 6–8 weeks were chosen and randomly separated into two groups (*n=5* each). 2 × 106 cells (MDA-MB-231-MCS and -GPRC5A) were injected subcutaneously into each mouse at the fourth mammary fat pad.

Tumor volume was measured once every 3 days and calculated by the formula: length × width^2^/2. All animal experiments were conducted according to the standard operating procedures approved by the Institute Research Ethics Committee of Tianjin Medical University Cancer Hospital.

Immunohistochemistry and TUNEL Assay. Immunohistochemistry and TUNEL staining were performed using standard protocols with paraffin-embedded mouse xenograft tumors. These tissues were stained using GPRC5A antibody (YT3995, immunoway, 1:1000). TUNEL staining was performed according to the manufacturer’s protocol (12156792910, invitrogen).

Statistical Analysis. GraphPad Prism 5.0 software (GraphPad Software, Inc.) was used for statistical analysis. Data are presented as the mean ± SD. Two-tailed Student’s t-test was used for comparisons between groups, while differences between tumor and adjacent normal control samples were analyzed using a paired Student’s t-test. For comparisons among multiple groups (≥3 groups), one-way ANOVA was used followed by Fisher’s least significant difference post-hoc test. P<0.05 was considered to indicate a statistically significant difference. All experiments were performed in triplicate.

## Results

**Figure 1:**
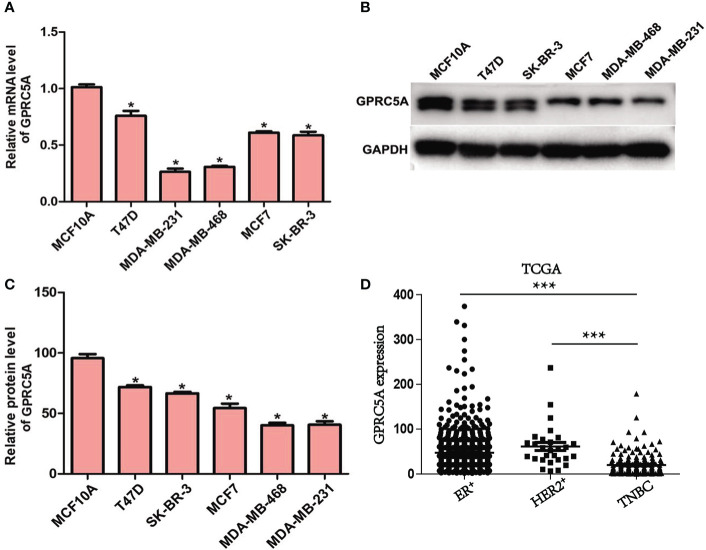
Expression of GPRC5A is downregulated in human breast cancer. **(A)** Relative mRNA expression levels of GPRC5A were examined in breast cancer cell lines and a control normal breast epithelial cell line using reverse transcription-quantitative PCR. **(B)** Protein expression levels of GPRC5A were examined in cultured human breast cancer cell lines and the control normal breast epithelial cell line MCF10A by western blot analysis. **(C)** Quantification of the western blot analysis results. ^*^P < 0.05 vs. MCF10A. GPRC5A, G-protein-coupled receptor family C, member 5, group A. **(D)** The expression of GPRC5A in TNBC was compared to ER^+^ and HER2^+^ breast cancer from TCGA database. ***P < 0.05.

GPRC5A Expression Is Downregulated in Human Breast Cancer Cells. First, the expression of GPRC5A in breast cancer cells was examined. The mRNA expression levels of GPRC5A were significantly lower in breast cancer cells compared with MCF10A control cells (**Figure 1**). Similarly, the protein expression levels of GPRC5A were significantly lower in breast cancer cells compared with the MCF10A (**Figure 1B****)**. Notably, MDA-MB-231 and MDA-MB-468 cells exhibited the lowest expression levels of GPRC5A, while T47D and MCF7 cells exhibited the highest mRNA expression levels of GPRC5A among the breast cancer cell lines investigated. Therefore, MDA-MB-231 and MDA-MB-468 cells were selected for subsequent overexpression experiments, and T47D and MCF7 cells were selected for knockdown experiments. Additionally, GPRC5A is significantly downregulated in TNBC breast cancer compared to ER^+^ and HER2^+^ breast cancer from TCGA database. (**Figure 1**). And Immunohistochemistry staining assay showed the similar results (**Figure S1**). In summary, the present data suggested that GPRC5A was downregulated in human breast cancer, especially in triple negative breast cancer.

**Figure 2:**
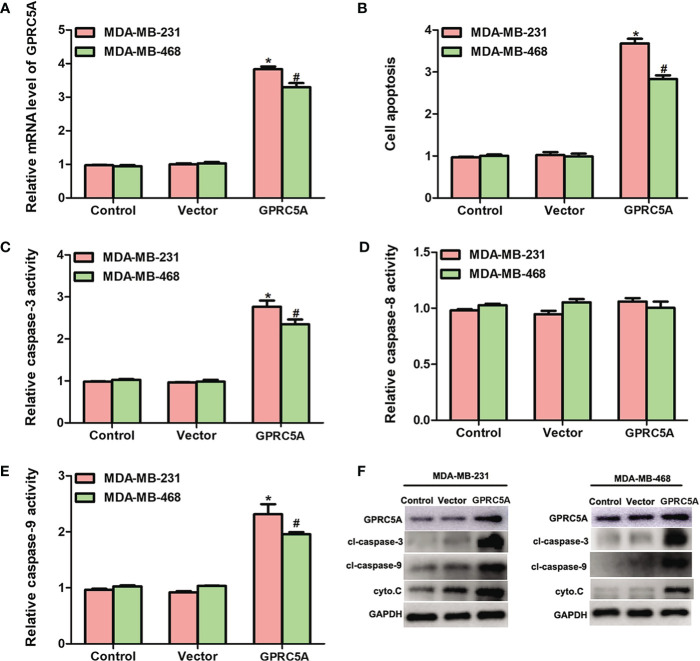
Overexpression of GPRC5A promotes cell apoptosis by increasing the activity of the intrinsic pathway. **(A)** Relative mRNA expression levels of GPRC5A in MDA-MB-231 and MDA-MB-468 cells transfected with a GPRC5A-overexpressing plasmid. **(B)** Cell apoptotic rates were assessed in MDA-MB-231 and MDA-MB-468 cells transfected with GPRC5A expressing plasmid. **(C)** Relative caspase-3 activity levels were examined in MDA-MB-231 and MDA-MB-468 cells transfected with GPRC5A-expressing plasmid. **(D)** Relative caspase-8 activity levels were evaluated in MDA-MB-231 and MDA-MB-468 cells transfected with GPRC5A expressing plasmid. **(E)** Relative caspase-9 activity levels were examined in MDA-MB-231 and MDA-MB-468 cells transfected with GPRC5A expressing plasmid. **(F)** Western blot analysis was performed to examine the levels of factors associated with the intrinsic pathway of cell apoptosis. ^*^P<0.05 vs. control in MDA-MB-231 cells. ^#^*P*<0.05 vs. control in MDA-MB-468 cells. GPRC5A, G-protein-coupled receptor family C, member 5, group A; cyto.C, cytochrome C; cl, cleaved.

Overexpression of GPRC5A Promotes Cell Apoptosis by Increasing the Activity of the Intrinsic Apoptotic Pathway. The detailed role of GPRC5A in human breast cancer was then investigated. GPRC5A-expressing plasmid was transfected into MDA-MB-231 and MDA-MB-468 cells. The relative mRNA level of GPRC5A was significantly increased in transfected cells compared with the control cells for both cell lines (**Figure 2**). In addition, the cell apoptotic rates were increased by 3.5- and 3-fold following transfection in MDA-MB-231 and MDA-MB-468 cells, respectively (**Figure 2**, **Figure S1**). Cell apoptosis includes two distinct pathways; the intrinsic pathway, where caspase-3 and 9 are the major factors involved in the process, and the extrinsic pathway, where caspase-8 serves a crucial role (14). Following overexpression of GPRC5A in MDA-MB-231 and MDA-MB-468 cells, the relative activities of caspase-3 (**Figure 2**) and caspase-9 (**Figure 2**) were significantly increased compared with the control cells; however, no significant difference was identified in the relative activity of caspase-8 (**Figure 2**). Furthermore, protein expression levels were detected using western blot analysis. The protein expression levels of cleaved-caspase-3, cleaved-caspase-9 and cytochrome C, key molecules involved in the intrinsic pathway of cell apoptosis, were higher in MDA-MB-231 and MDA-MB-468 cells transfected with GPRC5A-expressing plasmid compared with the control cells (**Figure 2**). The present results suggested that overexpression of GPRC5A increased cell apoptosis in MDA-MB-231 and MDA-MB-468 cells.

**Figure 3:**
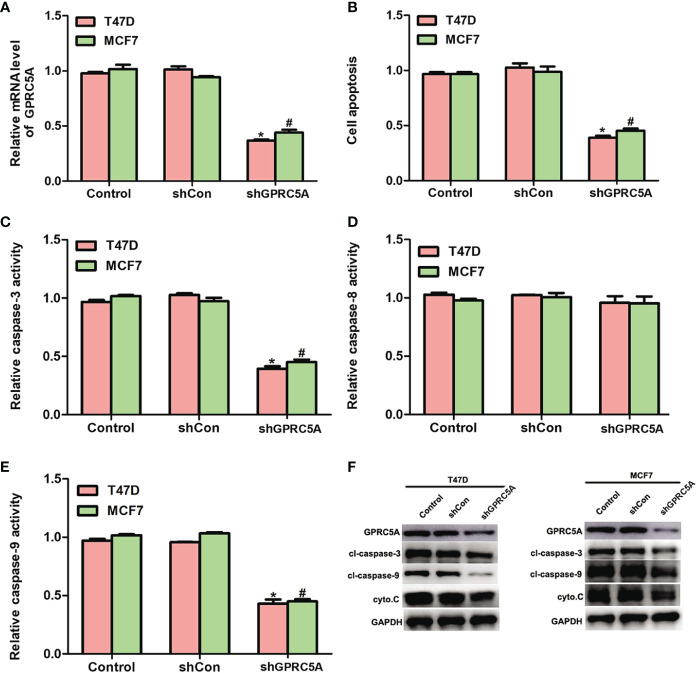
Knockdown of GPRC5A inhibits cell apoptosis by suppressing the activity of the intrinsic pathway. **(A)** Relative mRNA expression levels of GPRC5A in MDA-MB-231 and MDA-MB-468 cells transfected with shGPRC5A. **(B)** Cell apoptotic rates were assessed in MDA-MB-231 and MDA-MB-468 cells transfected with shRNA against GPRC5A. **(C)** Relative caspase-3 activity levels were examined in MDA-MB-231 and MDA-MB-468 cells transfected with shRNA against GPRC5A. **(D)** Relative caspase-8 activity levels were investigated in MDA-MB-231 and MDA-MB-468 cells transfected with shRNA against GPRC5A. **(E)** Relative caspase-9 activity levels were evaluated in MDA-MB-231 and MDA-MB-468 cells transfected with shRNA against GPRC5A. **(F)** Western blot analysis was performed to examine the levels of proteins associated with the intrinsic pathway of cell apoptosis. ^*^P < 0.05 vs. control in MDA-MB-231 cells. ^#^*P* < 0.05 vs. control in MDA-MB-468 cells. GPRC5A, G-protein-coupled receptor family C, member 5, group A; cyto.C, cytochrome C; cl, cleaved; shCon, small hairpin RNA control; shGPRC5A, GPRC5A small hairpin RNA.

Knockdown of GPRC5A Inhibits Cell Apoptosis by Suppressing the Activity of the Intrinsic Apoptotic Pathway. In addition, the present study performed knockdown of GPRC5A using a specific shRNA and investigated its effects on cell apoptosis. The relative mRNA levels of GPRC5A were significantly decreased in transfected cells compared with control cells (**Figure 3**). In addition, cell apoptosis was significantly suppressed by GPRC5A shRNA in both cell lines (**Figure 3**, **Figure S1**). The relative activities of different caspases were also detected, and the activity of caspase-3 (**Figure 3**) and caspase-9 (**Figure 3**) significantly decreased in the transfected cells compared with the control cells; however, no significant difference in the activity of capase-8 was detected in T47D and MCF7 cells (**Figure 3**). Similarly, the protein expression levels of cleaved-caspase-3, cleaved-caspase-9 and cytochrome C were markedly decreased when T47D and MCF7 cells were transfected with shGPRC5A for 48 h (**Figure 3**). The present data suggested that GPRC5A promoted cell apoptosis *via* the intrinsic apoptotic pathway in human breast cancer cells *in vitro*.

**Figure 4:**
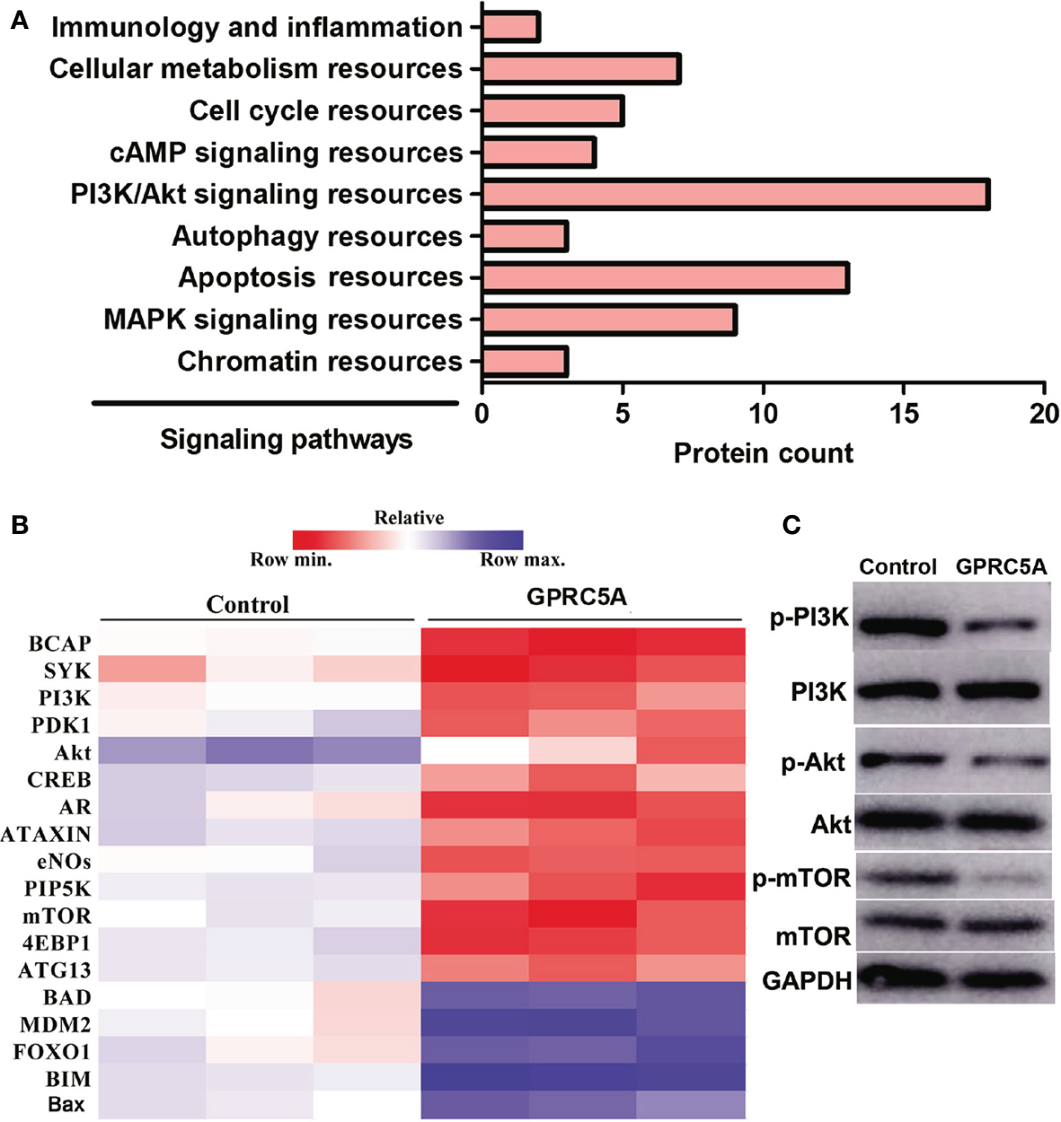
Overexpression of GPRC5A inhibits the PI3K/Akt signaling pathway in MDA-MB-231 cells. **(A)** RNA-sequencing analysis revealed the top nine signaling pathways associated with the GPRC5A regulatory network in MDA-MB-231 cells transfected with GPRC5A expressing plasmid. **(B)** Heatmap of the 18 differentially expressed proteins that are associated with the PI3K/Akt signaling pathway. Red indicates downregulated and blue indicates upregulated genes. GPRC5A, G-protein-coupled receptor family C, member 5, group A; p-, phosphorylated; MAPK, mitogen-activated protein kinase; BCAP, B-cell adaptor for phosphoinositide 3-kinase; PDK1, phosphoinositide-dependent kinase-1; CREB, CAMP responsive element binding protein; AR, androgen receptor; eNOs, endothelial nitric oxide synthase; PIP5K, phosphatidylinositol-4-phosphate 5-kinase; 4EBP1, eukaryotic translation initiation factor 4E-binding protein 1; ATG13, autophagy-related protein 13; MDM2, mouse double minute 2 homolog; FOXO1, forkhead box protein O1; BIM, Bcl-2-like protein 11. **(C)** Western blot analysis was performed to examine the levels of proteins associated with PI3K/Akt signaling pathway.

Overexpression of GPRC5A Inhibits the PI3K/Akt Signaling Pathway in MDA-MB-231 Cells. The detailed mechanisms underlying the regulatory role of GPRC5A in cell apoptosis were then investigated using RNA-seq techniques. Among all genes that were identified to be differentially expressed in MDA-MB-231 cells transfected with GPRC5A expressing plasmid, those with significant differential expression (P<0.05) were selected and their associated signaling pathways were determined. Notably, 18 genes differentially expressed in MDA-MB-231 cells following GPRC5A overexpression were identified to be associated with the PI3K/Akt signaling pathway (**Figure 4**). These 18 genes were presented in a heat map and it was identified that the expression levels of a number of genes, including B-cell adaptor for phosphoinositide 3-kinase, PI3K and Akt, were downregulated, while numerous genes associated with the PI3K/Akt signaling pathway, including Bad and Bax, were upregulated following overexpression of GPRC5A (**Figure 4**).

**Figure 5:**
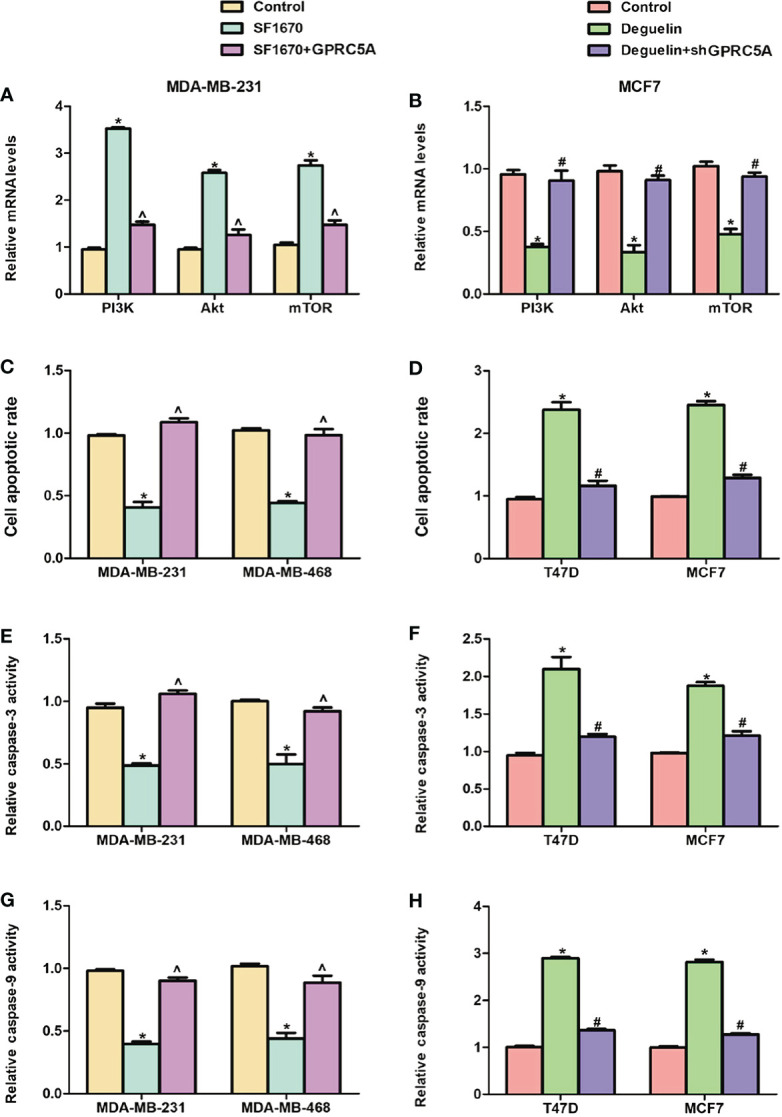
Interference of the PI3K/Akt signaling pathway reverses the effects of GPRC5A on cell apoptosis in breast cancer cells. **(A)** Relative mRNA expression levels of PI3K, Akt and mTOR in MDA-MB-231 cells treated with SF1670 in presence or absence of GPRC5A. **(B)** Relative mRNA expression levels of PI3K, Akt and mTOR in MCF7 cells treated with deguelin in the presence or absence of shGPRC5A. **(C)** Cell apoptosis was assessed in MDA-MB-231 and MDA-MB-468 cells following treatment with SF1670 with or without GPRC5A overexpression. **(D)** Cell apoptosis was assessed in T47D and MCF7 cells following treatment with deguelin with or without shGPRC5A. **(E)** Relative activity levels of caspase-3 were examined following treatment with SF1670 and GPRC5A-overexpressing plasmid in MDA-MB-231 and MDA-MB-468 cells. **(F)** Relative activity levels of caspase-3 were examined following treatment with deguelin and shGPRC5A in T47D and MCF7 cells. **(G)** Relative activity levels of caspase-9 were examined following treatment with SF1670 and GPRC5A-overexpressing plasmid in MDA-MB-231 and MDA-MB-468 cells. **(H)** Relative activity levels of caspase-9 were examined following treatment with deguelin and shGPRC5A in T47D and MCF7 cells. ^*^P<0.05 vs. corresponding control; ^#^*P*<0.05 vs. deguelin; ^^^*P*<0.5 vs. SF1670. GPRC5A, G-protein-coupled receptor family C, member 5, group A; sh, small hairpin.

Pharmacologic Interference of the PI3K/Akt Signaling Pathway Reduces the Effects of GPRC5A on Cell Apoptosis in Breast Cancer Cells. To elucidate the importance of the PI3K/Akt signaling pathway in the regulatory role of GPRC5A, the PTEN inhibitor SF1670 and deguelin were used to activate and inhibit, respectively, the PI3K/Akt signaling pathway. The present results suggested that MDA-MB-231 cells treated with SF1670 exhibited significantly increased mRNA expression levels of PI3K, Akt and mTOR, while cotreatment with SF1670 and GPRC5A-overexpressing plasmid significantly reduced this effect (**Figure 5**). Furthermore, when MCF7 cells were treated with deguelin, the mRNA expression levels of PI3K, Akt and mTOR were significantly decreased, while cotreatment with deguelin and shGPRC5A significantly restored the expression levels of these factors (**Figure 5**). Subsequently, cell apoptosis was examined, and MDA-MB-231 and MDA-MB-468 cells treated with SF1670 presented significantly reduced cell apoptosis (**Figure 5**, **Figure S2**), and decreased activities of caspase-3 (**Figure 5**) and caspase-9 (**Figure 5**). Notably, overexpression of GPRC5A significantly reversed these effects. Similarly, treatment of T47D and MCF7 cells with deguelin significantly increased cell apoptosis (**Figure 5**, **Figure S2**), as well as the activities of caspase-3 (**Figure 5**) and caspase-9 (**Figure 5**), while knockdown of GPRC5A significantly reversed these effects. In summary, the present results suggested that GPRC5A promoted cell apoptosis by regulating the PI3K/Akt signaling pathway in breast cancer cells.

**Figure 6:**
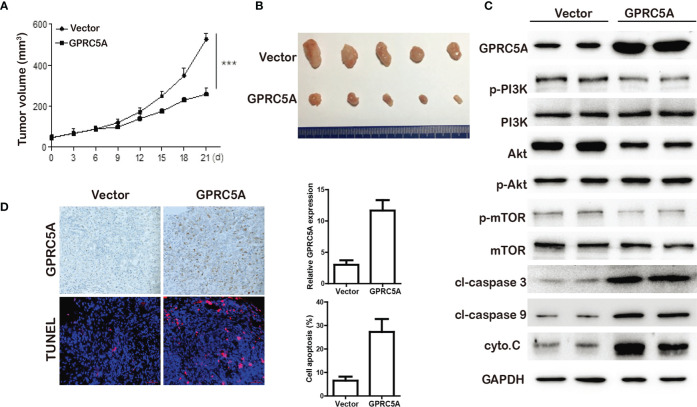
GPRC5A inhibited TNBC tumor growth *in vivo*. **(A)** The tumor volume were measured. ***P<0.05 vs. control. **(B)** Tumors from MDA-MB-231-MCS and MDA-MB-231-GPRC5A xenograft mouse model. **(C)** Western blot analysis was performed to examine the levels of proteins associated with cell apoptosis. **(D)** The expression of GPRC5A was examined by immunohistochemistry. The cell apoptosis was measured by TUNEL staining. *P<0.5, ***P<0.05 vs. control. GPRC5A, G-protein-coupled receptor family C, member 5, group A; cyto.C, cytochrome C; cl, cleaved.

GPRC5A Inhibited TNBC Tumor Progression In Vivo. To investigate the role of GPRC5A on TNBC tumor progression, the MDA-MB-231 mouse xenograft was established. Stable MDA-MB-231-MCS and MDA-MB-231-GPRC5A cells were injected subcutaneously. As shown in **Figure 6A**, GPRC5A significantly inhibited MDA-MB-231 xenograft tumor growth. The western blot assay showed that upregulated GPRC5A promotes the expression of cleavage-caspase 3, cleavage-caspase 9 and cytochrome C, the markers of apoptosis, *via* suppressing the activity of PI3K/Akt signaling pathway (**Figure 6**). Immunohistochemistry staining and TUNEL assay showed that the upregulation of GPRC5A promoted the apoptosis in mouse xenograft model (**Figure 6**). These results collectively demonstrated that GPRC5A leads to TNBC tumor progression *via* PI3K/Akt pathway *in vivo*.

## Discussion

Breast cancer is one of the most common types of malignancy in females worldwide and affects ~12% of females (15). Breast cancer cases contribute to 22.9% of invasive cancer cases and 16% of all cases of cancer in females (16). According to a European study, 523,000 new cases were diagnosed in 2018 and 138,000 breast cancer-associated mortalities occurred, making the incidence rate of breast cancer one of the highest of all cancer types, with a morbidity rate that ranked third among all cancer types (17). TNBC accounts for 15–25% of all breast cancer cases and the incidence rate is very similar among all age groups (18). Despite significant efforts in the past decades, the 5-year survival rate remains low. Therefore, there is an urgent requirement to identify novel therapeutic methods for the diagnosis and treatment of breast cancer. In the present study, GPRC5A was identified to be downregulated in breast cancer cells. Using an *in silico* assay, a previous study has suggested that GPRC5A is highly expressed in breast cancer, particularly in ER-positive breast cancer compared with the normal breast cancer cell line MCF-10A (19). However, the present results suggested that the expression of GPRC5A was decreased in cultured breast cancer cells, in line with a previous study (11). Other studies have reported that GPRC5A is an orphan G-protein coupled receptor with an intriguing dual behavior, acting as an oncogene in certain cancer types and as a tumor suppressor in other cancer types (20). GPRC5A-KO mice was highly associated with lung metastasis and poor prognosis (21, 22). In breast cancer, GPRC5A has been reported to be downregulated and was identified to act as a tumor suppressor by RhoA/C (23) and EGFR related signaling pathway (11). GPRC5A could be a malignant biomarker in breast cancer progression. Thus, the role and mechanism of GPRC5A in breast cancer, especially in different breast cancer subtypes, still need elucidated for further application of GPRC5A as a prospective clinical target.

Since cell apoptosis is an irreversible process, it is highly regulated (24). Apoptosis can be divided into two pathways; in the intrinsic pathway, apoptosis occurs following intracellular stresses, while in the extrinsic pathway, apoptosis occurs following extrinsic signals (25). In the intrinsic pathway, cytochrome C is released from the mitochondria and binds to apoptotic protease activating factor-1 and ATP, forming a complex that binds to pro-caspase-9 and forms an apoptosome, which cleaves pro-caspase-9 to its active form caspase-9, which activates the effector caspase-3 (26). In total, two theories of the extrinsic pathway have been suggested; the tumor necrosis factor-induced model and the Fas-Fas ligand-mediated model, the former of which involves the activation of caspase-8 (27). The present study identified that overexpression of GPRC5A promoted apoptosis and the activities of caspase-3 and caspase-9, while knockdown of GPRC5A inhibited these processes. These results suggested that GPRC5A affected cell apoptosis *via* the intrinsic pathway.

The PI3K/Akt signaling pathway is an intracellular signaling pathway that is directly associated with cell proliferation, tumorigenesis, cellular quiescence, longevity and apoptosis (28). Briefly, PI3K activation phosphorylates and activates Akt, leading to its translocation to the plasma membrane and the subsequent activation of its downstream genes, including CAMP responsive element binding protein, forkhead box O and mTOR (29). Using RNA-seq analysis, the expression levels of 18 genes associated with the PI3K/Akt signaling pathway were identified to be significantly altered following GPRC5A overexpression in MDA-MB-231 cells. Therefore, the PI3K/Akt signaling pathway was selected for further investigation. A number of factors can enhance PI3K/Akt signaling, including epidermal growth factor, insulin-like growth factor 1 and insulin (30). In addition, numerous factors can inhibit the PI3K/Akt signaling pathway, PTEN, glycogen synthase kinase 3 β and homeobox protein 9 (31). Therefore, the PTEN inhibitor SF1670 was selected as an activator of the PI3K/Akt signaling pathway and deguelin was selected as an inhibitor of PI3K/Akt signaling in the present study. The activation of PI3K/Akt pathway has been a focus of interest in breast cancer due to its role in cell growth, cell migration and deregulated apoptosis (32). The inactivation of PI3K/Akt pathway is an important approach in triple negative breast cancer (33). The present results suggested that the effects of SF1670 and deguelin were reversed by GPRC5A overexpression and knockdown, respectively. The present results supported the aforementioned hypothesis that GPRC5A can act as an upstream regulator of the PI3K/Akt signaling pathway.

In conclusion, the present study suggested that GPRC5A was downregulated in human breast cancer cell lines. Overexpression of GPRC5A promoted cell apoptosis by increasing the activity of the intrinsic pathway and inhibition of GPRC5A exhibited the opposite effect. GPRC5A was identified to regulate cell apoptosis *via* the PI3K/Akt signaling pathway. In summary, the present study identified GPRC5A as a potential protective factor against breast cancer progression and provided novel insights on the mechanism of GPRC5A on TNBC cell apoptosis.

## Data Availability Statement

The original contributions presented in the study are included in the article/**Supplementary Material**. Further inquiries can be directed to the corresponding author.

## Ethics Statement

The animal study was reviewed and approved by Institute Research Ethics Committee of Tianjin Medical University Cancer Hospital.

## Author Contributions

LY designed the study, performed the analysis, and wrote the paper. JZ supervised the work. TZ edited the paper. SZ made contribution to the revised manuscript. All authors contributed to the article and approved the submitted version.

## Funding

This study was supported by the National Natural Science Fund (No. 81672623) and Key Projects in Tianjin Science & Technology Pillar Program (No. 19YFZCSY00030).

## Conflict of Interest

The authors declare that the research was conducted in the absence of any commercial or financial relationships that could be construed as a potential conflict of interest.
