# Supplementary material for: GPRC5A Is a Negative Regulator of the Pro-Survival PI3K/Akt Signaling Pathway in Triple-Negative Breast Cancer
Source: Front Oncol. 2021 Feb 16;10:624493. doi: 10.3389/fonc.2020.624493 (PMC7928339; doi:10.3389/fonc.2020.624493)
Supplement: Supplementary file 1 [file DataSheet_1.docx]

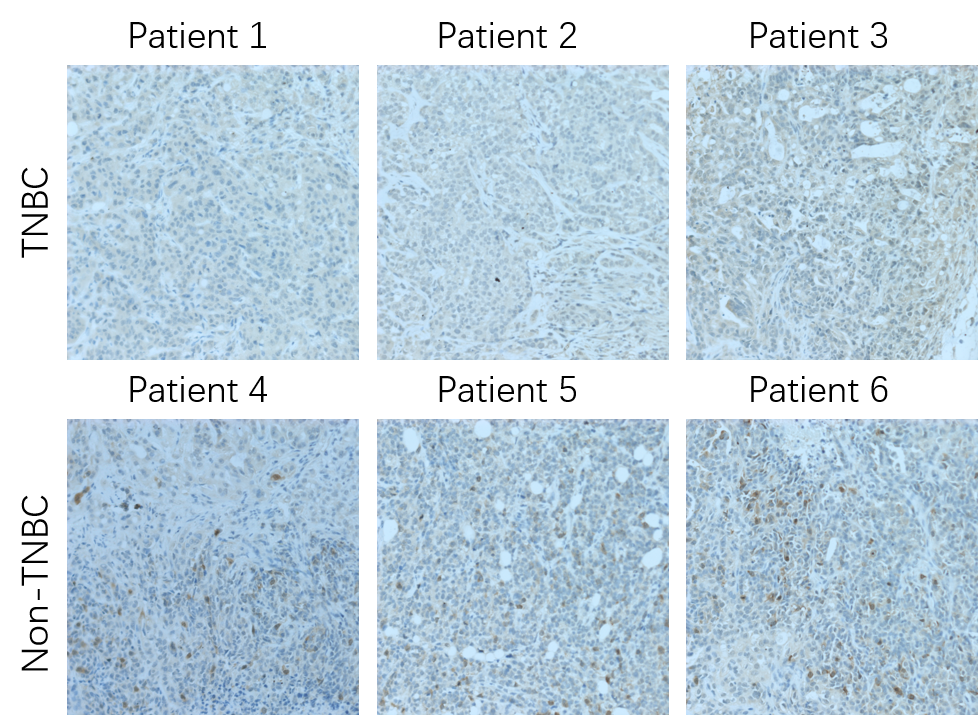


Figure S1 Immunohistochemistry staining assay of GPRC5A on non-TNBC and TNBC breast cancer tissues.


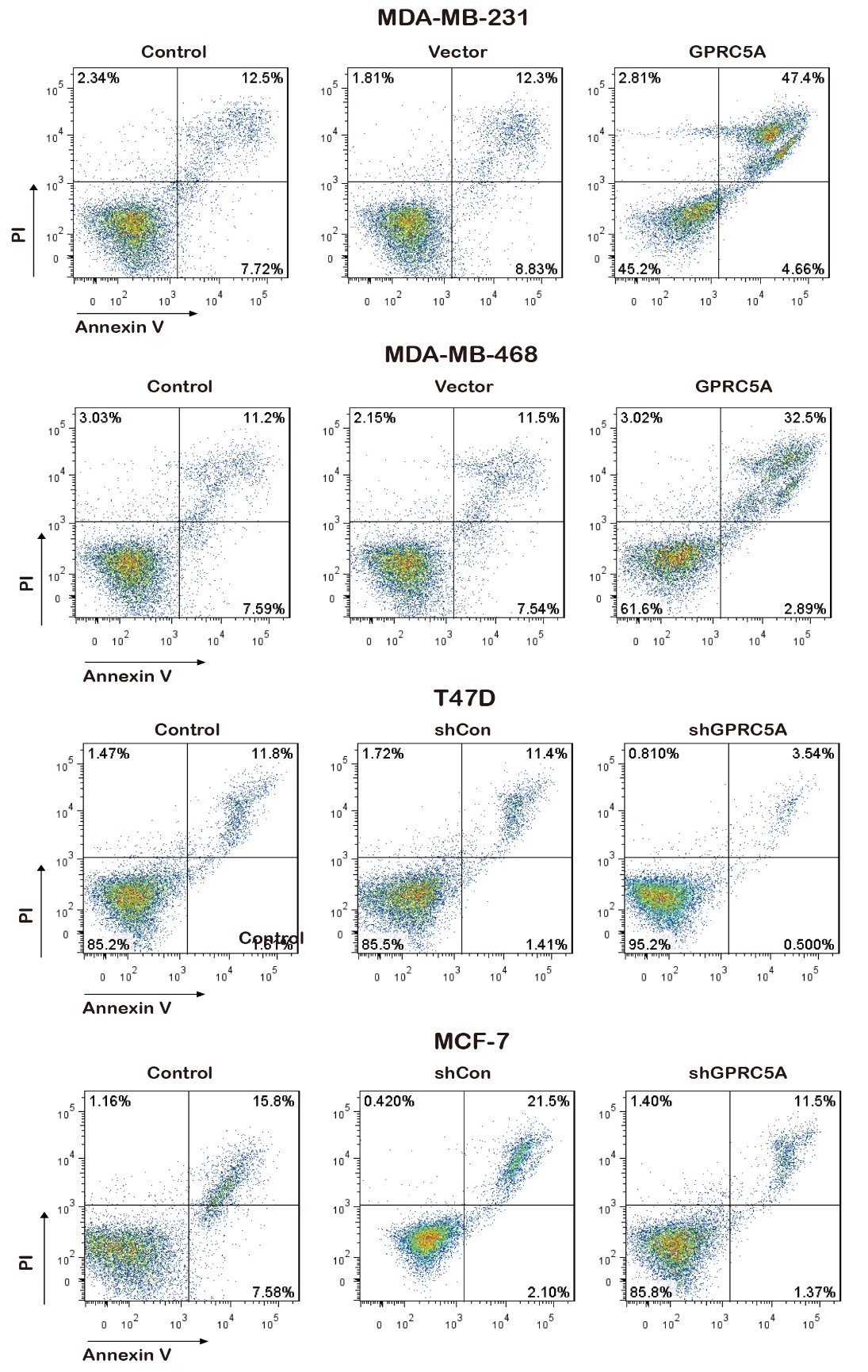


Figure S2 Flow cytometric analysis of cell apoptosis in MDA-MB-231/ MDA-MB-468/ T47D/ MCF-7 transfected with vector control (Vector/ shCon), GPRC5A or shGPRC5A. The control group is wildtype cells.


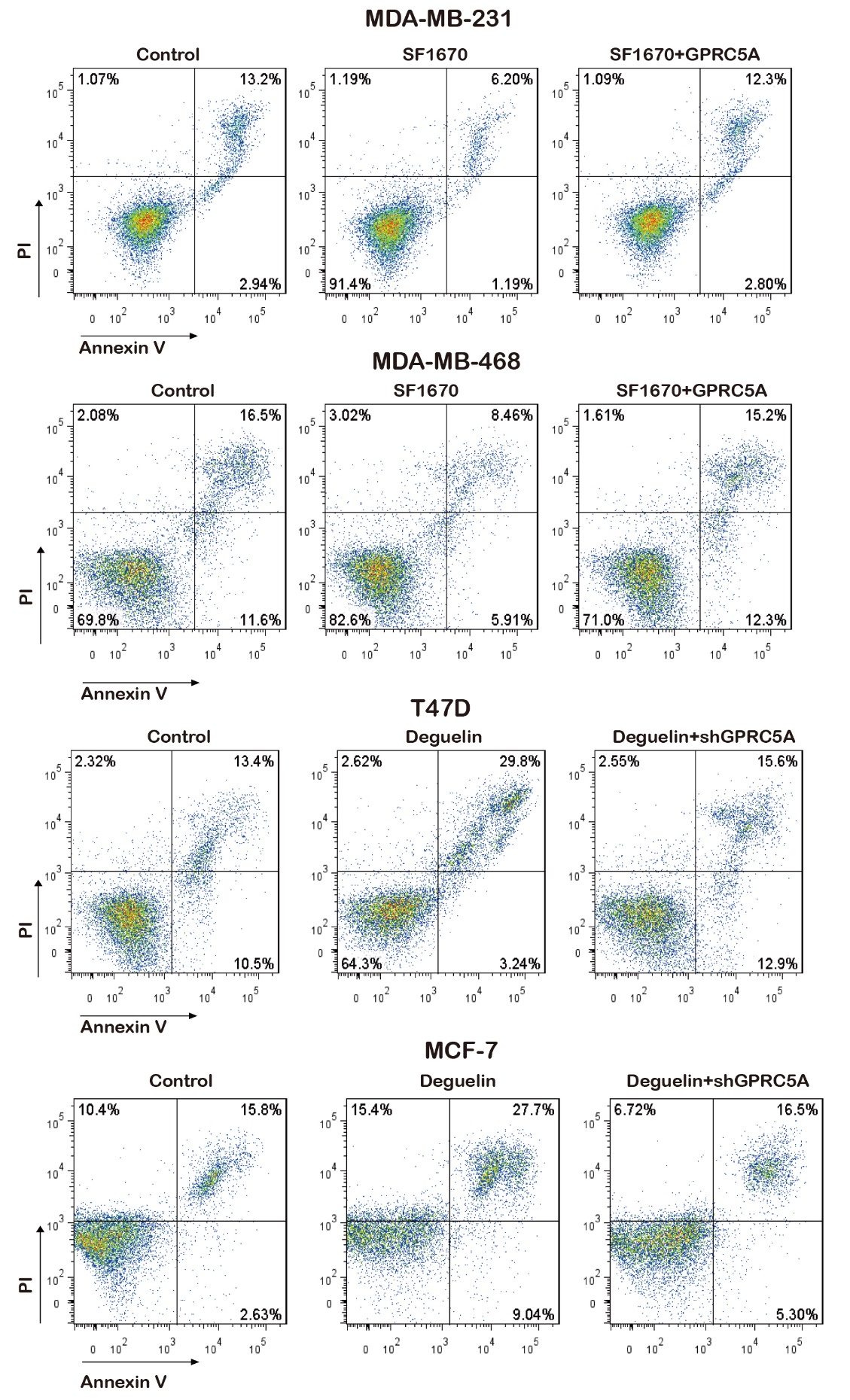


Figure S3 Flow cytometric analysis of cell apoptosis in MDA-MB-231/ MDA-MB-468/ T47D/ MCF-7 transfected with vector control (Vector/ shCon), GPRC5A or shGPRC5A following treatment with SF1670 or Degurlin. The control group is wildtype cells.
